# The Main Molecular and Serological Methods for Diagnosing COVID-19: An Overview Based on the Literature

**DOI:** 10.3390/v13010040

**Published:** 2020-12-29

**Authors:** Bruna Aparecida Souza Machado, Katharine Valéria Saraiva Hodel, Valdir Gomes Barbosa-Júnior, Milena Botelho Pereira Soares, Roberto Badaró

**Affiliations:** 1 SENAI Institute of Innovation (ISI) in Health Advanced Systems (CIMATEC ISI SAS), University Center SENAI/CIMATEC, Salvador 41650-010, Bahia, Brazil; katharine.hodel@fbter.org.br (K.V.S.H.); valdir.junior@fieb.org.br (V.G.B.-J.); milena.soares@fieb.org.br (M.B.P.S.); badaro@fieb.org.br (R.B.); 2 Gonçalo Moniz Institute, Oswaldo Cruz Foundation (IGM-FIOCRUZ/BA), Salvador 40296-710, Bahia, Brazil

**Keywords:** SARS-CoV-2, COVID-19, diagnostic, molecular testing, serology testing

## Abstract

Diagnostic tests have been considered as the main alternative for the control of coronavirus disease (COVID-19), caused by the severe acute respiratory syndrome coronavirus 2 (SARS-CoV-2), as a correct diagnosis allows for decision making when facing the disease, particularly as there is a lack of effective therapeutic protocols and vaccines. Thus, in this review, we summarized the main diagnostic approaches currently available for the diagnosis of SARS-CoV-2 infection in humans based on studies available in article databases. The tests can be organized into two main categories: nucleic acid-based tests, recommended for the initial detection of the virus, and serological tests, recommended for assessing the disease progression. The studies have shown that the performance of diagnostic methods depends on different factors, such as the type of samples and the characteristics of each assay. It was identified that the positivity of the tests is mainly related to the onset of symptoms. We also observed that point-of-care diagnoses are considered as one of the main trends in this area, due to the low-cost and simplicity of the assay; however, the analytical performance must be critically analyzed. Thus, the COVID-19 pandemic has highlighted the critical role of diagnostic technologies in the control of infectious diseases.

## 1. Introduction

The battle against infectious diseases caused by microorganisms, particularly viruses, remains a challenging and endless task despite the enormous forces and significant advances in public healthcare from different parts of the world [1]. Due to the advances of medicine, various researchers and scientists believed that the battle of humanity against infectious agents was virtually over, with humankind as the winners [2]. However, the repeated outbreaks of the past two decades including coronaviruses [3], avian influenza [4], and chikungunya [5], have shown the prematurity of that position. Recently, at the end of 2019, a novel fast-spreading respiratory disease was reported in Wuhan, in the Hubei province of China, and has now affected over 216 countries globally [6,7]. The complete viral genome data suggested that the disease is caused by a new RNA virus related to the family *Coronaviridae*, which was later designated as severe acute respiratory syndrome coronavirus 2 (SARS-CoV-2). The disease was later named as novel coronavirus disease (COVID-19).

Coronaviruses (CoVs) are large sized (100–160 nm), spherical, enveloped, non-segmented, positive-sense RNA viruses, known to be broadly distributed in humans and other mammals [8]. CoVs genome are the largest RNA viral genome, ranging from 26 to 32 kb in length. Typically, two thirds of the genomic RNA encodes for two large overlapping polyproteins, open reading frame (ORF) 1a and ORF1b, which are processed into the viral polymerase (RdRp) and other nonstructural proteins (Nsps) involved in RNA synthesis or host response modulation [9]. The other third of the genome encodes four structural proteins (spike (S), envelope (E), membrane (M), and nucleocapsid (N)) and other accessory proteins (3a, 3b, p6, 7a, 7b, 8b, 9b, and ORF14) [10]. Genome-wide phylogenetic analysis indicated that SARS-CoV-2 shares 80% and 50% sequence identity with SARS-CoV and Middle East Respiratory Syndrome Coronavirus (MERS-CoV), respectively, which are other important coronaviruses that were responsible for previous endemics [11,12]. Additionally, SARS-CoV-2 presents more than 90% sequence identity for essential enzymes and structural proteins, indicating a common pathogenesis mechanism and, therefore, a common therapeutic targeting with these viruses [11].

Individuals infected with SARS-CoV-2 may present with symptoms, such as fever, dry cough, fatigue, or shortness of breath, with or without nasal congestion, runny nose, or other respiratory symptoms [13]. However, patients with mild symptoms may not present any symptoms or can be minimally symptomatic [14]. In addition, asymptomatic patients are also able to transmit SARS-CoV-2 via respiratory droplets (aerosols), which has contributed to the fast increase in the number of COVID-19 cases worldwide [15].

Rapid and accurate detection of the causative pathogen is essential in controlling the outbreak among both asymptomatic carriers and individuals showing signs of the disease [16], as identification of carriers can break the trains of community transmission and allow for contact tracing and providing timely treatment [17]. Thus, epidemiologists consider mass testing for SARS-CoV-2, which requires a high test rate per inhabitant, as the most practical route out of the current outbreak, as it allows officials to isolate those who presents a positive diagnosis, help to determine when it is safe to relax restrictions, and control the spread of SARS-CoV-2, since there were only one recently approved vaccine (vaccine BNT162b2 received emergency use approval from United Kingdom health authorities) [18] or specific drug therapies against SARS-CoV-2 available at the time of this review [19].

By the end of September, the United States was the country that performed the highest number of diagnostic tests to detect SARS-CoV-2 (more than 110 million), followed by India (more than 71 million), Russia (more than 45 million), and the United Kingdom (more than 19 million) [20]. Brazil and Mexico are among the 10 countries with the highest number of reported COVID-19 cases [6], but failed to occupy the same position when it comes to the number of tests per every 1000 inhabitants [20]. The low-test rate per inhabitant indicates that the number of infected persons in these countries may be even higher, which may make it difficult to take strategic measures to combat the pandemic.

Various diagnostic methods have been proposed to provide a rapid response in the combat against the pandemic, each of them having a different degree of specificity and based on different target molecules from SARS-CoV-2 or the body in response to infection [21,22]. These methods include molecular and serological tests [23], as well as clinical tests, such as computed tomography (CT) [24], and each approach has its own advantages and shortcoming. Thus, the diagnostic methods present different performances depending on the type of the test and the stage of the disease, among other factors [25,26].

For example, the molecular technique of quantitative reverse transcription–polymerase chain reaction (RT-qPCR) is considered the “gold standard” for identification of SARS-CoV-2 and has been recommended by different health agencies [27,28]; however, its low performance in different studies suggests that the diagnosis for COVID-19 may be better given by using combined techniques [29,30]. RT-qPCR helped some countries in Asia, such as Korea and Singapore, to initially control the outbreak caused by SARS-CoV-2 [31,32]. However, the limitation of high prices and lengthy procedures impede the use of these assays as mass testing for middle-income countries [33]. Many studies are currently being conducted to determine the prevalence of antibodies against SARS-CoV-2 in certain populations, which has increased the demand and use of serologic tests [34,35,36].

Therefore, in view of the relevance presented by the diagnostic tests, as they are still considered the most important measure to combat the COVID-19 pandemic, we summarized the main diagnostic approaches currently available for diagnosis of SARS-CoV-2 infection in humans based on studies available in article databases, in particular, considering the performance of each type of test and the main challenges and perspectives related to each one.

## 2. Nucleic Acid-Based Tests

The advance in molecular biology technology has allowed for the development of nucleic acid-based detection assays that have become an important technology and the most widely used method for virus detection [1,37,38]. During this ongoing COVID-19 outbreak, nucleic acid detection has played an important role in early diagnosis. The molecular techniques utilize the genetic material of the virus and are based on the principle of the specificity of base pairing with homologous strands. From the genetic sequencing of SARS-CoV-2 [11], different genetic material-based detections have been proposed, including methods based on polymerase chain reaction (PCR) [39], isothermal nucleic acid amplification (loop mediated isothermal amplification (LAMP) [40,41], and clustered regularly interspaced short palindromic repeats (CRISPR) [42,43]. When compared with other available tests, these methods are far more sensitive, and can detect viruses much earlier in clinical samples [44].

### 2.1. Quantitative Reverse Transcription–Polymerase Chain Reaction (RT-qPCR)

**Figure 1:**
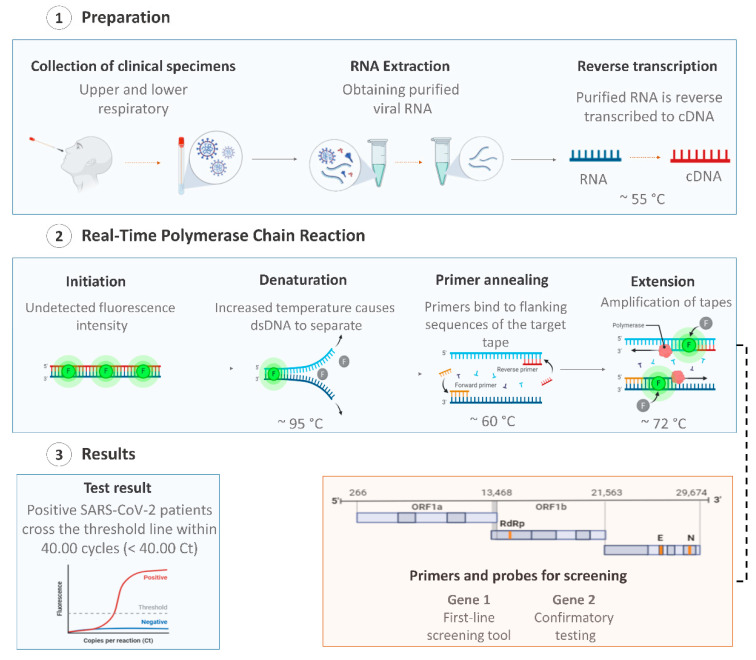
Overview of coronavirus disease (COVID-19) diagnosis using the quantitative reverse transcription–polymerase chain reaction (RT-qPCR) technique with respiratory tract specimens. Created with BioRender.com.

The viral nucleic acid-based test using quantitative reverse transcription–polymerase chain reaction (RT-qPCR) is the first line screening method of choice for SARS-CoV-2 detection [45], being regarded as the “gold standard” test due to its high sensitivity, rapid detection, and other desirable characteristics [46,47]. Additionally, the RT-qPCR technique is the most suitable method, as it allows for viral detection and quantification [48,49]. RT-qPCR tests are used for the identification and differentiation of SARS-CoV-2 in the samples/specimens collected from symptomatic and asymptomatic patients, by detecting specific RNA sequences of SARS-CoV-2 [50]. In general, these tests involve three essential steps: (i) extraction of viral RNA from the collected specimens; (ii) reverse transcription of viral RNA to a single-stranded DNA (cDNA) using the enzyme reverse transcriptase; and the final step (iii) the amplification of the cDNA coupled to fluorescent detection (Figure 1) [51].

RT-qPCR is a versatile test and can be used to detect the virus in a various range of specimens, such as saliva, sputum, lower respiratory tract secretions, blood, urine, stool, nasopharyngeal, and oropharyngeal [16,45,52,53]. Since the disclosure of the first SARS-CoV-2 genome sequence, various candidate diagnostic RT-qPCR assays were designed and made available in the public domain. Agencies and manufacturers have chosen specific and different sets of genes, and RTq-PCR commercial kits are currently widely available on the world market [51]. Therefore, those tests differ in sensitivity, stability, accuracy, and assay time [7].

Studies on SARS-CoV-2 are still evolving and the extent of the virus mutations is still not clear [54]. An ideal nucleic acid test design should include a conserved region and a specific region of the viral genome to minimize against the effects of SARS-CoV-2 genetic drift as the virus evolves within different populations [55,56]. The first RT-qPCR for SARS-CoV-2 detection was developed by China’s Center for Disease Control and Prevention (CDC) and was designed to detect the N and the ORF1ab genes (Table 1) [57]. In this test, the infection is confirmed when both markers are amplified [27]. However, sometimes, the results from the two pairs of primers do not agree with each other and the result needs to be re-tested [58].

The same recommendation is made by the United States (US) CDC, where, if only one target is positive in the N region target, the result is considered inconclusive and need to be re-tested [27]. Thus, all two or three regions tested should be positive to identify a positive case [58]. The first RT-qPCR protocol developed outside of China incorporated primers targeting genes of the E, N, and RdRp and was developed by the Charité Institute [48].

Regarding other protocols, Vogels et al. [57] examined the sensitivity and efficiency of four common RT-qPCR assays developed by the US CDC, China CDC, Germany Charité, and Hong Kong University (HKU). This study found that, while all primer-probe sets could detect SARS-CoV-2, the most sensitive primer-probe sets were the Germany E gene, HKU-ORF1, HKU-N, and US CDC N [57]. While the US CDC N2 primers had background cross-reactivity, this did not interfere with the outcomes of the combined N1 and N2 assay when testing clinical samples from COVID-19 patients [57]. In addition, other studies have also compared the US CDC N1 and N2 against commercial test kits, reporting similar specificities with variable sensitivities [59,60]. Therefore, the performance of RT-qPCR tests can be evaluated according to molecular targets (Table 1).

In this context, a retrospective study of RT-qPCR test results for 4880 patients suspected of SARS-CoV-2 infection utilizing the ORF1ab and N protein gene fragments of viral as targets, reported that 1875 patients had a positive test result with a low test sensitivity of 38.42% on samples from the respiratory tract [61]. In another study, the researchers developed one-step RT-qPCR assays to detect ORF1b and N regions of SARS-CoV-2, where the N gene assay was used for screening, and ORF1b was used as confirmatory test [62]. However, the authors reported that the assay involved the detection of ORF1b and N regions that are highly conserved among sarbecoviruses, and therefore it may also bind to SARS-CoV and other closely-related viruses, which may result in a cross-react.

Another approach assessed the performance of RT-qPCR assays targeting the RdRp/helicase (Hel), S and N genes and compared it to an RdRp-P2 assay [56]. Among them, the COVID-19-RdRp/Hel assay was significantly more sensitive in detecting the presence of SARS-CoV-2 compared with the RdRP-P2 assay, where the positive rate for the RdRp/Hel assay was 43.6% and for the RdRP-P2 assay was 28.2% (*p* < 0.001) [56]. The genes E and RdRp were the most commonly used to detect the COVID-19 virus as both have high analytical sensitivity (technical limit of detection of 3.2 and 3.6 copies per reaction, respectively) [44,48,63].

Unlike OFR1b and N, the E and RdRp genes are more conserved in the SARS-CoV-2 genome, which leads to a high performance of the test and, consequently, a lower probability of cross-reactivity with other homologous viruses [2,63]. Thus, the test design is essential to guarantees double confirmation in cases of viral infection as it decreases the risk of obtaining false negative results in the case of detection of only one target for SARS-CoV-2 [51].

An important factor for the application of the real time RT-qPCR technique is the type of platform and kit used. Currently, with the broad access to RT-qPCR kits, the ability to diagnose the population increases and helps in combating the pandemic. Some kits have received emergency use authorization from the United States Food and Drug Administration (FDA) or other regulatory agencies, while others only report validations made by manufacturers, and in general little is known about their performances [7,64]. These tests are most often performed in large labs at commercial diagnostic facilities or academic centers. Specimens from individuals with suspected infection must, therefore, be sent to these labs, increasing both the time and coordination requirements [65]. Thus, different alternatives have been proposed for improvements in the performance of the RT-qPCR assay with the use of high throughput platforms (or systems), which involve an automated workflow to enable high throughput testing with minimal hands-on time, and still offering fast, reliable results [66].

The automated molecular diagnostic platform is a highly sensitive and accurate alternative for the rapid identification of SARS-CoV-2 [67]. An important RT-qPCR rapid test is the Xpert Xpress SARS-CoV-2 test (Cepheid, Sunnyvale, CA, USA), which is able to provides results in less than 1 h using the GenXpert benchtop system [68]. Table 2 presents a performance analyses of studies related to RT-qPCR kits and different platforms for the diagnosis of SARS-CoV-2.

Significant differences were noticed in the viral loads of specimens depending on where in the respiratory tract they were collected, which could interfere with the RT-qPCR test performance [69,70]. Li et al. [71] compared the detection rate of SARS-CoV-2 by RT-qPCR in throat swabs and sputum specimens of 52 patients with pneumonia caused by SARS-CoV-2, based on diagnostic criteria from the China CDC. The results showed that the type of sample interfered with the accuracy of the test, where the detection rates of SARS-CoV-2 from sputum specimens were significantly higher than those from throat swabs using the assay, at 76.9% and 44.2%, respectively [71].

The results reported in the study by Wang et al. [45] showed that a positive rate was 93% in bronchoalveolar lavage fluids, 72% for sputum, 63% for nasal swabs, and 32% in pharyngeal swabs after analysis by RT-qPCR. Other studies indicated that different viral load kinetics of SARS-CoV-2 interfered with the accuracy and sensitivity of the RT-qPCR test, since, depending on the specimen timing and period of the disease, the results may be false negative or false positive [72,73,74]. Therefore, they suggested that specimen timing and the period of the disease, as well as the specimen’s location and development play an important role in RT-qPCR results.

In addition, there are few studies that tested the RT-qPCR method in a control group. Among them, the study by Xie et al. [75] tested the RT-qPCR method in a control group. In trial, the specificity was 100% for stool, urine, blood, nasal swab, and throat swab samples, while throat swab and sputum samples had specificities of 98.6% [75]. The control group allowed the researchers to perform statistical analyses on the parameters of specificity, positive likelihood ratio, and negative likelihood ratio [63].

Changes have occurred in the protocols of health agencies and health surveillance regarding the number of swabs collected to perform RT-qPCR testing for the detection of SARS-CoV-2. At the beginning of the pandemic, it was recommended that two swabs per patient be used for upper respiratory tract sampling. However, due to a lack of materials, mainly swabs, currently agencies recommend that the collection be performed with only one swab for the two nostrils of the patient [28,76]. No studies were found to assess the relevance of this change with respect to the test performance.

The performance of the RT-qPCR test can be influenced by different factors, which can result in low sensitivity, stability, and accuracy. More specifically, the low accuracy of RT-qPCR kits may be less than optimal (i.e., <100%) resulting in the growth of false negative results, especially among asymptomatic patients [77,78]. Studies have shown the high rate of false-negative results in different countries of Asia using the RT-qPCR test [47,79]. A study reported a case of severe COVID-19 with an initial seven consecutive false-negative RT-qPCR results in the First Affiliated Hospital, School of Medicine, Zhejiang University, also in China [80]. In addition to the Asian continent, a study in the Principality of Liechtenstein, located in Europe, showed that the rate of false-negative RT-qPCR reached 18% [81]. False-negative results may occur by mutations in the primer and probe target regions in the SARS-CoV-2 genome [82]. False negatives can bring problems associated with the control of COVID-19, as this prevents the diagnosis of the disease in patients, and it also risks patients who assume that they are not infected but can transmit the virus to the community [2].

Based on these limitations of RT-qPCR tests, different studies proposed that the diagnosis of COVID-19 be carried out by associating imaging techniques, such as CT [93,94]. CT, more specifically chest CT, has been an important imaging modality in the diagnosis and management of patients with viral pneumonia, as demonstrated in the outbreaks in SARS-CoV [95], MERS-CoV [96], and currently with SARS-CoV-2 [97]. However, the diagnosis of COVID-19 by RT-qPCR remains indispensable due to its benefits, and the chest CT can be considered as a supplemental tool for diagnosing COVID-19 in symptomatic and asymptomatic patients [98]. Thus, the combination of RT-qPCR and clinical features facilitates the management of the SARS-CoV-2 outbreak.

### 2.2. Loop Mediated Isothermal Amplification (LAMP)

Isothermal amplification technology was developed to eliminate the need for a high-precision instrument in RT-qPCR assays [66]. This technique is commonly used for the amplification of DNA and RNA and exhibits high sensitivity due to its exponential amplification feature and specificity as a result of the six different target sequences identified by four different primers simultaneously [99,100]. A LAMP positive reaction can be determined visually and quantified based on fluorimetry, colorimetry, and turbidity.

For this, the insoluble byproduct (magnesium pyrophosphate) formed during the LAMP reaction can be seen with the naked eye (cloudiness) [101]. Thus, the LAMP method is fast and does not require expensive reagents or instruments, such as a thermal cycler, unlike RT-qPCR [102]. Another important factor is that the LAMP assay is considered a point-of-care (PoC) diagnostic tests (i.e., a cost-efficient and rapid assay that can be performed without robust equipment and that does not necessarily require a trained technician to operate) diagnostic test [103,104]. In the current scenario, PoC tests are crucial and urgently needed for the detection of SARS-CoV-2. A reverse transcriptase step is included in the LAMP reaction (RT-LAMP) to allow RNA targets to be detected. The technique was further developed to enable RNA detection by reverse transcription with successful application in the detection of numerous RNA viruses, including H7N9 influenza virus [103], Zika virus [104], MERS-CoV [105], and SARS-CoV [106].

Unlike the RT-qPCR, there are not many kits for SARS-CoV-2 detection by RT-LAMP technique released by regulatory agencies, such as the US FDA. Among them, the ID Now COVID-19 (Abbott, Chicago, Illinois, USA) is the most used, being able to detect the presence of SARS-CoV-2 in only 5 min [107]. However, ID Now showed a lower sensitivity when compared to the RT-qPCR test [108]. Thus, studies have been focused on comparative analysis between techniques to prove the RT-LAMP performance and that it can be useful in clinical laboratories to support the preliminary detection of SARS-CoV-2 in suspected patients [40,109,110]. 

Jiang et al. [111] demonstrated a rapid RT-LAMP assay that allows the processing of 2–2.5 more clinical samples relative to the CDC RT-qPCR protocol. Huang et al. [112] developed four sets of LAMP starters (comprising six starters in each set/kit) focused on viral RNA of SARS-CoV-2 in the regions of the ORF1ab, S gene, and N gene. Another study also analyzed the colorimetric method for interpretation of the ORF1ab target gene amplification results from 248 respiratory samples from patients positive to COVID-19 (RT-qPCR verified) [113]. The results showed that the tested method, named iLACO (isothermal LAMP based method for COVID-19), had a sensitivity of 89.9% (223/248) [113].

Another approach was reported by Yan et al. [40], who evaluated the RT-LAMP assay for detection of ORF1ab and S genes of SARS-CoV-2 (fluorescent detection) in comparison to RT-qPCR of 130 swabs and bronchoalveolar lavage fluid samples obtained from individuals with pneumonia and suspected SARS-CoV-2 infection. The results showed that the sensitivity and specificity of both methods were 100% [40]. The sensitivity of RT-LAMP for SARS-CoV-2 using upper and lower respiratory tract specimens was reported as equivalent to RT-qPCR in other studies [113,114]. However, the results reported in another study indicated that the sensitivity of RT-LAMP was inferior to RT-qPCR in saliva specimens; thus, it is necessary to pay attention to the false-negative results of RT-LAMP [41]. Thus, the performance of the RT-LAMP assay can be influenced by factors similar to the RT-qPCR method. A variation of the RT-LAMP method is LamPORE™, which combines the RT-LAMP technique with rapid preparation of barcoded libraries and real-time nanopore sequencing to create a way to rapidly test and screen a large number of samples for rapid, accurate and highly scalable detection. The differential of this technique is that a PCR step is not necessary for the nanopore sequencing, removing amplification bias, and consequently optimizing the process [115].

The main challenges of the RT-LAMP method are in designing sequence-specific primers and optimizing the reaction conditions. Within this context, the difficulty of primer design and their multiplicity might lead to non-specific amplification, causing inaccurate results [101]. With the spread of SARS-CoV-2, the accuracy of this RT-LAMP assay with an established set of primers may be affected by mutations occurring in the sequence region of the target gene. Thus, it is necessary to periodically monitor the mutant sites of the viral genome by whole-genome sequencing [40]. Another limitation associated with the RT-LAMP test is that false-positive amplification due to hybridization by primers or nontarget sequences can be common [116,117]. To this problem, a possible solution is to detect the specific sequences of the amplification products [117].

### 2.3. Clustered Regularly Interspaced Short Palindromic Repeats (CRISPR)

The prokaryotic defense system named “clustered regularly interspaced short palindromic repeats” (CRISPR) machinery, which utilizes segments of nucleic acid containing short, repetitive base sequences and enzymes (Cas), has been exploited for genome editing, known as CRISPR-Cas9, as well as CRISPR-Cas12a, and CRISPR-13 [118]. Recent insights into the biology of CRISPR/Cas led to new molecular diagnostics that leverage the activity of Cas effectors for sensitive, specific, and rapid nucleic acid detection [119].

The Cas12 or Cas13 proteins are guided by a CRISPR RNA (crRNA) to target a specific nucleic acid sequence, while the single-stranded region of the crRNA is complementary to the target. The functions of Cas12 and Cas13 are different: Cas12 targets ssDNA, while Cas13 targets ssRNA [117]. CRISPR-Cas-based assays can be run directly on primary clinical samples as a single reaction and performed using minimal equipment [42,120]. Therefore, like the LAMP assay, CRISPR is also considered as a PoC diagnostics for COVID-19 testing [65,121]. Lateral flow readouts can be coupled to CRISPR for reading the results, making it an attractive option for at-home testing scenarios [119].

Recently, the FDA granted Sherlock Biosciences, the manufacturers of the SHERLOCK (specific high-sensitivity enzymatic reporter unlocking) platform, an emergency use authorization for CRISPR SARS-CoV-2 rapid diagnostic (Sherlock CRISPR SARS-CoV-2 Kit). This authorization is an important milestone in the molecular area, since Sherlock CRISPR SARS-CoV-2 test is the first authorized use of CRISPR technology for an infectious disease test [122,123]. The CRISPR-Cas13 based SHERLOCK COVID-19 detection protocol searches for unique nucleic acid targets (SARS-CoV-2 ORF1ab and S genes) and uses a dipstick as the visual readout in less than an hour [124].

In addition to the SHERLOCK platform protocol, other protocols based on CRISPR for detecting SARS-CoV-2 have been published, such as DETECTR (DNA endonuclease-targeted CRISPR trans reporter) [125] and CRISPR-FDS (CRISPR-based fluorescent detection system) [42]. Both protocols reported promising results with high analytical sensitivity and, therefore, their application has the potential to improve the current COVID-19 screening efforts. The DETECTR diagnostic platform has also been used with the LAMP technique [125].

### 2.4. Perspectives and Challenges Associated with Molecular Diagnosis

The molecular techniques have revolutionized diagnostic virology by detecting the presence or absence of viral nucleic acids in a patient’s samples. These assays are becoming essential for the initial diagnosis of COVID-19, helping to conduct the clinical treatment of the disease. In addition to the tests already mentioned (RT-qPCR, RT-LAMP, and CRISPR), other molecular techniques can be used for the detection of SARS-CoV-2, such as microarray assays and viral sequencing (next-generation sequencing); however, their use is still restricted [126]. The current capacity of testing cannot meet unprecedented global demand for rapid molecular diagnosis. As presented in the previous topics, these techniques have limitations that can directly influence the real quantification of infected people and, consequently, increase the risk of transmission. Thus, although positive molecular results are indicative of active infection with SARS-CoV-2, negative results do not exclude SARS-CoV-2 infection.

Even with the analytical performance being widely questioned, RT-qPCR testing is still recommended as first choice of suspected cases by government or society guidelines in different countries. The Canadian guidelines are mostly concordant with the WHO recommendations for primary screening of suspected cases by RT-qPCR [127], as well as India [8], Brazil [128], Portugal [129], and China [130]. However, in Iran, a shortage of RT-qPCR testing kits led local medical authorities to establish an imaging-driven screening system, with chest CT representing the first-line diagnostic modality [131], even though there were only 6.5 CT scanners per million population [132]. The shortage of RT-qPCR kits has not only been reported in middle-income countries but also in certain states of the United States at times when there were peaks in the number of COVID-19 cases [133].

The measure adopted in Iran highlighted one of the main challenges of molecular techniques, such as RT-qPCR, microarray, and sequencing: the complexity of the necessary steps for the execution of the assays, which goes from collecting specimens to using complex equipment. These steps require important logistical decisions to be made for the supply of all necessary materials (e.g., nasopharyngeal swabs or viral transport media) and the allocation of equipment in restricted and appropriate sites (preferably biosafety level 2 or above). In addition, the need for specific equipment, as well as the consumables, present a high acquisition cost. For example, a single RT-qPCR test kit may cost over US$ 100, while the set-up of a diagnostic/processing lab requires more than US$ 15,000 [134]. In times of health system crises, such as the current one caused by the COVID-19 outbreak, waiting for supply of materials and the training of people for the correct operation of the equipment, as well as the cost of the molecular tests can be considered as important disadvantages.

Even with the high cost associated with using the RT-PCR technique, Neilan et al. [135] observed that individual testing of people with any symptoms consistent with COVID-19 could save costs compared to testing only on individuals whose symptoms justify hospital care. The study also shows that expanding RT-PCR testing to asymptomatic individuals could decrease infections, deaths and hospitalizations, which could be cost-effective in all epidemic scenarios [135]. However, mass testing using the RT-PCR technique can be a major challenge for populous countries, especially those with few financial resources. It is currently estimated that population-wide testing on large populations will be possible through pool-based strategies [136]. The basic principle behind the pooling approach is based on the mixing of a pre-selected number of samples in a batch [137]. In this way, the pooled (or combine) sample will be tested by applying a molecular diagnostic protocol, usually RT-qPCR, and the individual test is only necessary if a specific pool presents a positive result [138]. A pooled testing strategy allows a reduction in the costs associated with the use of molecular techniques as it reduces the total number of tests. Therefore, the pooled-based methods may be considered as an interesting alternative for low-to-middle-income countries [139], since the financial challenges are a limiting factor for the expansion of the number of tests per inhabitant in these regions.

In addition, PoC diagnostic tests, such as those based on RT-LAMP and CRISPR techniques, can potentially minimize financial and logistic limitations, due to: (1) a lack of sophisticated equipment; (2) fast genetic material amplification and detection; (3) minimal specimen handling, avoiding operator exposure to the virus and maintaining the integrity of the sample; (4) tests easily performed by personnel without complex training; and (5) the time of analysis being reasonably short [117]. Thus, diagnostic tests with this approach are considered as the big bet for the coming years. However, despite the advantages cited, it is essential that these tests have their performance properly validated, as their sensitivity may be variable due to the more rapid nature of the test. Therefore, research and development validation studies should be highly prioritized in the next few months/years [65].

## 3. Serological Tests

The immune system produces antibodies in response to viral infections as a natural response of the human body against viral infection. This process, also known as the humoral response, is utilized for the development of immunological diagnostic methods [140]. Serology-based antibody tests can promote an estimate of the SARS-CoV-2 incidence, which can complement the nucleic acid-based tests, as they can detect individuals with immunity against the disease by markers of the immune response [141]. These tests have become increasingly relevant as the pandemic progresses, since it is extremely important to evaluate the antibody prevalence of the population that has already been exposed to the virus, either in cases of infection or due to immunization therapies.

When as individual has compatible clinical syndrome for COVID-19, evidence for disease with a clear exposure history to the pathogen and supportive laboratory and radiographic findings but has negative results by RT-qPCR test for SARS-CoV-2, a positive serologic assay can help to confirm the diagnosis and support and guide clinical management decisions [142]. More specifically, serologic testing enables the understanding of how patients produce antibodies to SARS-CoV-2, and assays can detect immunoglobulin A (IgA), IgM, IgG, or the total antibodies [65]. IgG is often the most abundant antibody in the blood and has as important role in the later stages of SARS-CoV-2 infection and in support of establishing long-term immune memory, while IgM antibodies are produced by host immune cells during the early stages of infection [143].

However, although IgM and IgG antibodies are the main targets of serological assays, IgA, predominately present in the mucosal tissue, may also play a critical role in the immune response and disease progression, can then be considered a target for detection [144]. Typically, the samples used for this type of assay are plasma, serum, or whole blood [26]. Thus, serological samples have less variations compared to nasopharyngeal or other respiratory specimens because antibodies are usually homogeneously dispersed in the blood [2]. Therefore, serologic assays are often unable to distinguish a current infection from a prior infection, and an important aspect is that the sensitivity and specificity of individual assays may vary drastically depending on the survey population [142].

In public health practice, serological analysis can be useful for rapid case detection and sequential events to identify nearby contacts and determine case groups and quarantines [141]. In addition, these tests are essential to understanding the immune response against SARS-CoV-2. There are three major types of serological diagnostic tests: enzyme-linked immunosorbent assays (ELISA), chemiluminescence immunoassays (CLIA), and rapid diagnostic tests (RDT).

**Figure 2:**
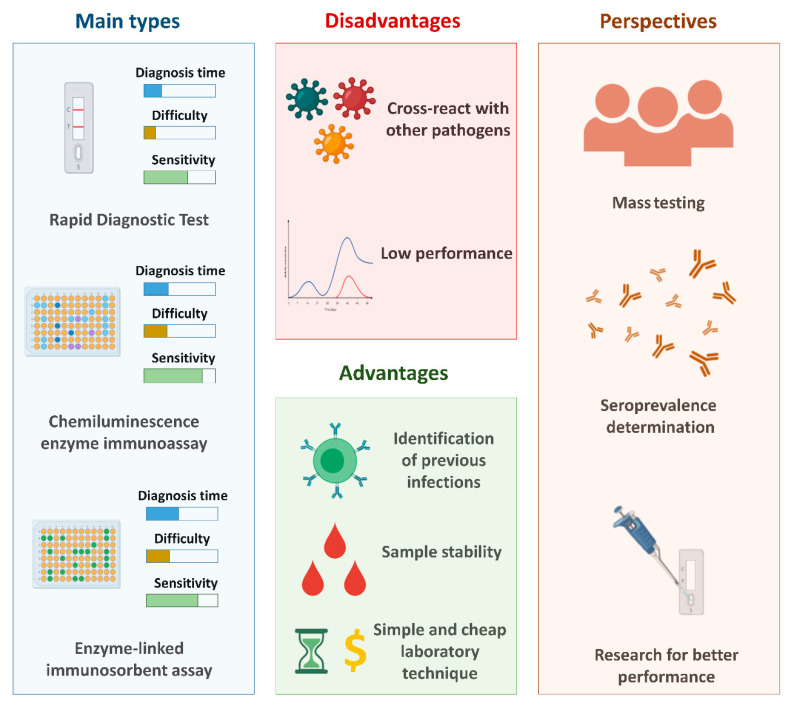
The main serological tests used for the diagnosis of COVID-19 and the main advantages and disadvantages related to these methods and their perspectives. Created with BioRender.com.

In general, compared to the gold standard test for the detection of SARS-CoV-2 (RT-qPCR), serological tests are considered less expensive, as they are cheaper and the diagnostic time is shorter. In addition, the steps involved in performing the tests are less complex, since they do not require steps related to the treatment of the clinical sample (such as the extraction step). Figure 2 shows the general properties of the main types of serological tests, in addition to the main advantages and disadvantages related to the use of these techniques and the perspectives associated to these tests.

### 3.1. Enzyme-Linked Immunosorbent Assay (ELISA)

**Figure 3:**
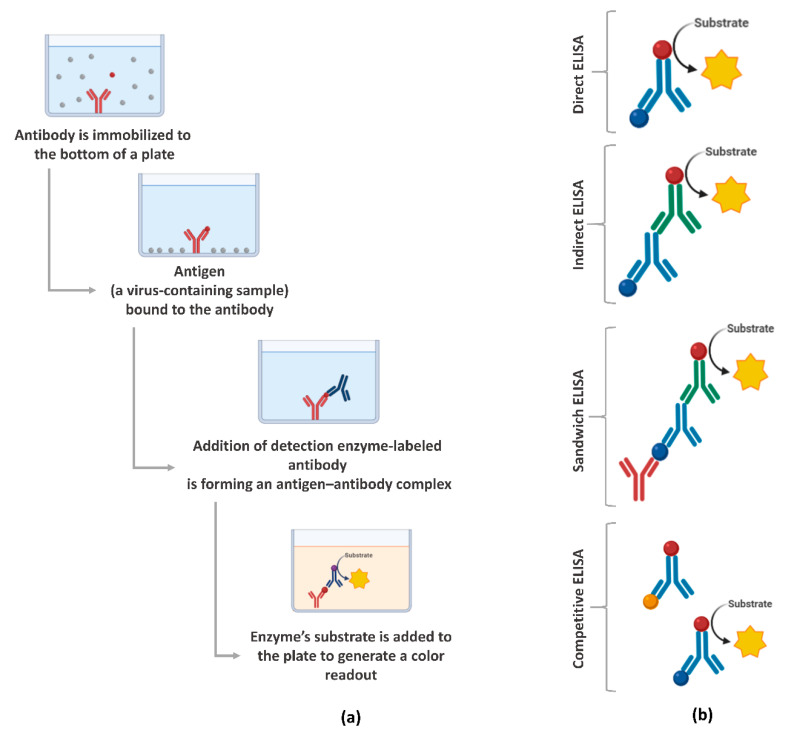
Enzyme-linked immunosorbent assays (ELISA) overview: (**a**) main steps for the execution of the assay and (**b**) the main formats. Created with BioRender.com.

ELISA is a plate-based assessment method for detecting and quantifying biomolecules, including proteins, such as hormones and antibodies or peptides [66]. ELISA assays can be found in different formats, including direct, competitive, and, the most commonly used, sandwich or double-antigen-bridging assay (DABA) [145]. Figure 3 presents an overview of the steps involved in the ELISA test and the main formats. In the context of the COVID-19 pandemic, the ELISA test has been widely used because it has a shorter time-to-results, when compared to RT-qPCR, and the ability to scale to very large throughput and simplicity [146].

Normally, commercially available ELISA kits are able to detect IgA, IgG, IgM, or IgG and IgM [147]. An ELISA test with combined IgM and IgG detection demonstrated overall increased sensitivity as evident from a study where 82.5% and 44.4% sensitivities were observed for IgG and IgM, respectively, when independently evaluated, whereas, in combination, the sensitivity increased to 87.3% and the specificity was 100% [148]. In addition, the combination of the ELISA test along with other techniques for the diagnosis of COVID-19 was also used to decrease the cases of false negative or positive results. Guo et al. [149] demonstrated that the detection rate by RT-qPCR was higher than the IgM ELISA before 5.5 days post–symptom onset, while the detection efficiency by IgM ELISA was higher than that of the RT-qPCR method after 5.5 days of symptom onset. In general, the positive detection rate was only 51.9% in a single RT-qPCR test, but significantly increased (98.6%) when an IgM ELISA assay was applied to PCR-negative patients.

In addition to the tests developed for the detection of IgA, IgM, and IgG there are also some commercially available ELISA kits for detecting SARS-CoV-2 viral antigens (spike and nucleocapsid proteins); however, these are mainly used in research and not for clinical diagnosis [107]. Liu et al. [150] compared the sensitivity of recombinant SARS-CoV-2 nucleocapsid protein (rN) and spike protein (rS) for SARS-CoV-2 IgM/IgG detection via an ELISA method in 214 serum samples from confirmed cases by RT-qPCR. The study reported that 68.2% and 70.1% of COVID-19 patients were successfully diagnosed with the rN protein-based IgM and IgG ELISAs respectively, and 77.1% and 74.3% were diagnosed using the rS protein-based IgM and IgG ELISAs. However, a study by Zhong et al. [151] showed that N and S protein-based ELISA showed better sensitivity to the N protein compared with the S protein for IgM and IgG detection.

When using N protein-based ELISA assay, it may produce false positive results, as the nucleocapsid protein is the most conserved viral protein among human betacoronaviruses [152]. Due to this performance, the companies/manufacturers have focused on developing ELISA assays for detecting serum antibodies against two domains in the S protein (S1 and S2) [54]. Thus, the performance of the ELISA assay can be related to the progression of the disease and protein-based assays for the antibody. Table 3 presents the performance analyses of studies related to serological ELISA kits for the diagnosis of SARS-CoV-2.

Another important point when it comes to the performance of ELISA tests is related to the quantification of neutralizing antibodies. Mazzini et al. [153] observed that the RBD-based ELISA was efficient for quantifying IgA, IgM and IgG neutralizing antibodies against SARS-CoV-2. However, the study by von Rhein et al. [154] showed that although the ELISA is capable of quantifying neutralizing antibodies, its performance was lower when compared to the plate reduction neutralization test (PRNT) and a lentiviral vector based pseudotype neutralization assay. The low performance of this assay in this context may be related to its ability to detect only neutralizing antibodies that block the interaction between the RBD region and the receptor-angiotensin converting enzyme II (ACE2) and, even though most neutralizing antibodies have this action, other types of neutralization mechanisms have been described [155].

In addition, the cross-reactivity with other antibodies is a major challenge to serological tests, including the ELISA assay [156]. Antigens used in this ELISA may react with antibodies against other types of coronavirus (HKU1, 229E, OC43, and NL63) that are known to cause the common cold [157]. Although many challenges exist, serology testing using ELISA offers great benefits as a therapeutic option to control the current pandemic and possible re-emergence of coronavirus and other emergent viruses in the future.

### 3.2. Chemiluminescence Immunoassay (CLIA)

CLIA is one of the most popular immunology assays in identifying infectious diseases and is considered as a fully automated variation of the standard ELISA. CLIA is an assay that combines the chemiluminescence technique with immunochemical reactions. In this assay, an antigen is coated on the surface of the test plate and the antibodies present in the sample bind to the antigens fixed, forming an antigen-antibody complex. After, a chemiluminescence (luminescent molecule) is used as the indicator [164]. This technique has some advantages, including reproducibility, cost effectiveness, and fast and precise measurement of the IgG and IgM antibody levels, as well as the ability to perform more clinical tests for other biomarkers, such as C-reactive protein (CRP), which also needs to be monitored in COVID-19 suspects [107,165].

The CLIA assay made an important contribution to the understanding of the antibody response (antibody kinetics) during the course of COVID-19 [166]. In a study by Jin et al. [167], the dynamic variance of IgM and IgG antibodies in COVID-19 patients, retrospectively, were reported, where the amount of IgM antibodies decreased over time as the IgG antibody concentration increased. More specifically, another study showed that the maximum concentration of antibodies was found to be 73.6% on days 16–18 for IgM and 98.6% on days 19–21 for IgG after the onset of symptoms [168].

Similar to the ELISA, the combined detection of IgM and IgG antibodies also increased the performance of the CLIA assay when compared to individual detection. Cai et al. [169] observed that the combination of IgM and IgG showed 81.52% positivity in comparison to 57.2% for IgM and 71.4% for IgG, with 100% specificity to the healthy controls and other diseases evaluated. In this study, the authors developed a peptide-based magnetic chemiluminescence enzyme immunoassay (MCLIA) to detect SARS-CoV-2 antibodies against ORF1a/b, N, and S proteins [169]. Similarly, another study demonstrated that the sensitivities and specificities of IgA, IgM, and IgG tests with PCR-confirmed patients were 98.6%, 96.8%, and 96.8% and 98.1%, 92.3%, and 99.8%, respectively, and after combining the three antibodies, the sensitivity and specificity increased to 99.5% and 100%, respectively [170]. The same study showed that the diagnostic accuracy based on RBD outperformed those based on nucleocapsid protein.

The type of chemiluminescence analyzer (system) can also influence the performance of the CLIA assay. In this context, four chemiluminescence immunoassay systems were analyzed, and the results showed that test performance differed according to the system, with a maximum sensitivity and specificity of 92% and 99.23% with the total antibody [171]. Among them, Diazyme DZ-Lite 3000 Plus (Diazyme Laboratories, Poway, CA, USA) and MAGLUMI CLIA (Snibe, Shenzhen, China) analyzers are the most popular systems for CLIA assay. These systems are capable of processing the samples in an automated and fast mode, while the DZ-Lite 3000 Plus Diazyme is capable of performing 50 tests per hour, and MAGLUMI CLIA can detect IgG and IgM in patient samples in 30 min [108].

The study by Montesinos et al. [172] demonstrated that, compared with the Euroimmun IgG/IgA ELISA assay, the Maglumi™ IgG/IgM CLIA assay showed a lower overall sensitivity, with 84.4% for IgG and 64.3% for IgM. However, both assays exhibited very similar specificities of IgG, which were 99% and 100% for the ELISA and CLIA assays, respectively. Comparing the CLIA assay with other techniques, another study demonstrated the superiority of antibody testing over nucleic acid testing (RT-qPCR) in moderate, severe, and critical COVID-19 cases, in a retrospective and observational study with 133 patients from the city of Wuhan [173].

For moderate cases, the positivity for IgM and IgG was 79.55% and 83.18%, respectively, higher than RT-qPCR (65.91%), whereas severe cases showed 82.69% and 100% positive ratios for IgM and IgG, respectively, in comparison to 71.15% of RT-qPCR. The superiority of CLIA was also demonstrated in the evaluation of critical cases, where the positive ratios were found to be 72.97% and 97.30% for IgM and IgG, respectively, in comparison to RT-qPCR with 67.57%. These studies demonstrated the importance of a combined laboratory diagnosis, as the techniques used may present different performances, which may lead to a false diagnosis.

### 3.3. Rapid Diagnostic Tests (RDT)

RDT based on immunochromatography (IC) or lateral flow immunoassays (LFIA) have expanded widely, adapting the immunoassay concept to be disposable, inexpensive, fast (around 15 to 60 min turnaround time), and qualitative (yes/no) and, thus, can be considered for point-of-care testing [7]. These tests are based on the capillary action of a membrane (usually a nitrocellulose membrane) that is able to capture and detect antibodies. To read the result, gold nanoparticles or other colored nanoparticles generate colored lines on the membrane if the analyte of interest is present in the sample (often a finger prick blood drop) [174].

Rapid lateral flow assays provide the advantage of a fast time to results, can be used in a non-laboratory environment, require a low sample volume (10 or 20 µL), and are of relative low-cost, in addition to being considered as one of the most important PoC diagnostic tests. Currently, most of the commercially available tests are on LFIA technologies for detecting human IgM/IgG antibodies [43]. In addition, this assay is considered as ideal for primary healthcare workers for the rapid testing of COVID-19 suspected cases.

In settings where challenges with molecular testing exist or access to laboratories is scarce, rapid serology tests offer a needed additional option. Due to this, RDT has become very popular, particularly in countries with restricted financial resources. For example, the Brazilian government declared, at the beginning of the pandemic in the country, the distribution of more than 10 million units of rapid kits [175]. In parallel, more than 50% of the tests initially registered by the Brazilian Health Regulatory Agency for the diagnosis of COVID-19 were rapid serological tests [176]. In Brazil, as in other countries, the acquisition of this test was directed to the monitoring of the epidemiology of COVID-19 among healthcare workers, one of the groups most exposed to the disease [176,177].

It is important to highlight that the clinical accuracy of rapid tests needs to be stringently evaluated before they are authorized for the mass screening of COVID-19. Some reports from many European countries, such as Spain, Italy, Czech Republic, Netherlands, and the United Kingdom, suggest that most of the rapid tests for COVID-19 procured from China did not show good analytical performance [178,179,180]. This has caused the sensitivity and specificity of this type of diagnostic test to be questioned, which contributed to the reduction of its analytical credibility among specialists when compared to the beginning of the pandemic.

A study by Adams et al. [181] observed that the performance of nine different commercially available LFIA devices was inadequate for most individual patient applications when compared to ELISA, as the overall sensitivities of LFIA tests after 10 days of disease onset ranged from 55% to 70% compared to RT-qPCR with 95–100% specificity. Two other studies from different continents, Germany and Iran, specifically, demonstrated a low sensitivity of the LFIA test for the diagnosis of COVID-19, where the values found were 47.9% and 36.4%, respectively [182,183]. However, Whitman et al. [184] demonstrated that ten LFIA tests were validated with RT-qPCR positive samples and found a 81.8–100% sensitivity and 84.3–100% specificity after 20 days of disease onset. Even though their analytical performance has been questioned, the diagnostic kits made in China are still the most used within the context of the COVID-19 pandemic.

Like CLIA and other techniques, different authors suggested a combined diagnosis between LFIA and other techniques to mitigate the chances of false positive and negative results. Imai et al. [185] observed that the LFIA assay had low sensitivity during the early phase of infection, and thus the LFIA assay alone is not recommended for initial diagnostic testing for COVID-19. If RT-qPCR is not available, the combination of chest CT and IC assay may be useful for diagnosing COVID-19.

Shen et al. [186] reported that LFIA was found to be a useful test to complement existing PCR-based assays for confirmation of COVID-19, and a delayed specific IgM antibody response was observed among COVID-19 patients with severe progression. The results reported above, as well as the studies presented in Table 4, demonstrate that the performance of the LFIA test depended mainly on the type of kit used and the day of disease onset. LFIA has, nevertheless, played an important role in the fight against coronavirus, as different countries have carried out mass testing for surveillance within their communities, helping to determine antibody prevalence of SARS-CoV-2 [33]. Through this determination, vulnerable groups can be identified, as was shown in the study by Hallal et al. [35], which highlighted the SARS-CoV-2 antibody prevalence in indigenous populations of Brazil using RDT. The authors show that high seroprevalence, combined with comorbidity, such as metabolic and cardiovascular diseases that are also rapidly increasing among Brazilian Indians, may impact the increased risk of death due to COVID-19 [35]. Thus, from this population-based data, control policies can be adopted and public resources can be allocated to mitigate the effects of the pandemic on these populations, in addition to promoting the rational use of this type of test [187].

### 3.4. Antigenic Tests

Antigen assays were recently introduced as a diagnostic tool to screen the spread of the COVID-19 pandemic in the population [197]. In general, antigen tests are immunoassays that are capable of detecting the presence of a specific viral antigen, which implies a current viral infection, unlike assays that detect antibodies, as previously presented. Another important difference is that this type of test may be performed with nasopharyngeal or nasal swab specimens [198]. To date, the number of antigen-based diagnostic tests is lower than those available for antibody detection [199]. The antigenic assays are not restricted to a particular format [7], such as the use of ELISA and chemiluminescent immunoassay, both for the detection of SARS-CoV antigens [200,201]. However, considering the current moment, the main focus is the use of the LFIA technique for diagnosis of COVID-19 because of its low cost and practicality [202].

Among them, BinaxNOW COVID-19 Ag Card POC SARS-CoV-2 (Abbott, USA) is a test based on LFIA for the qualitative detection of N protein antigen to SARS-CoV-2 from nasal swab specimens [203]. Perchetti et al. [204] reported that the BinaxNOW COVID-19 Ag CARD has an analytical sensitivity similar to the RT-qPCR test. Another important point is that the U.S. government has purchased some 150 million BinaxNOW antigen tests from Abbott, demonstrating a trend to use this type of approach for mass testing [203].

However, other studies show that, compared to molecular technology, rapid antigen detection tests were less sensitive than RT-PCR [205,206]. The study by Blairon et al. [207] showed that the rate of false negative results obtained through rapid SARS-CoV-2 antigenic testing was approximately 22%. In addition, since antigen tests have a lower specificity than RT-qPCR tests, high false positive rates may occur in low prevalence scenarios [202]. Therefore, negative results obtained through this rapid antigen detection method cannot exclude the real possibility of infection with the SARS-CoV-2. Recently, US FDA approved under emergency use authorization different rapid antigen tests [199,204]. However, it is important to emphasize that the use of these tests for clinical diagnosis or mass testing must be carefully established, since their performance must be improved.

### 3.5. Perspectives and Challenges Associated with Serological Diagnosis

Serological tests play a fundamental role in determining the epidemiology of disease and, in vaccine development, can provide a determination of antibody production and concentration months or years after infection, as well as antibody abundance and diversity [7]. The use of serological tests for mass testing has been considered as an interesting alternative, since it is important to determine the prevalence of antibodies against SARS-CoV-2, as well as through epidemiologic studies. In this way, knowledge about population groups with positive serological results can support governments in determining strategic health measures.

These tests have several intrinsic limitations that are mainly related to the individual’s immune response to the pathogen, in this case SARS-CoV-2, as well as the performance related to each type of kit according to the type of manufacturer. The vast majority of available serological kits assays (ELISA, CLIA, and RDT) measure antibody binding to SARS-CoV-2 spike protein. Since not all spike binding antibodies can block viral infection (neutralization), these assays do not functionally measure the inhibition of antibodies from SARS-CoV-2 infection [208]. Thus, other tests are used for the quantification of neutralizing antibodies, such as the “gold standard” PRNT; however, for the correct execution of this assay some important points are required, such as cell culture facilities, and in the case of SARS coronavirus, Biosafety Level 3 (BSL3) laboratories, as well as the time to results is typically 3–5 days [7]. This is an important gap for COVID-19 surveillance and vaccine development [208], since the quantification of neutralizing antibodies can support in determining the immunogenicity of a new vaccine or in treatment of passive immunization with convalescent plasma.

Within this context, the main challenges related to serological testing include: (1) most serological tests currently available for COVID-19 (especially the PoC antibody tests) do not measure neutralizing antibodies and, therefore, cannot conclude whether a previously infected person has sufficient neutralizing antibodies for immunity; and (2) many of the PoC antibody tests have poor clinical specificity, leading to a high rate of false positive and false negative results [117]. Therefore, the results from serological tests alone should not be used to diagnose SARS-CoV-2 infection in practice [2].

To mitigate these challenges, some measures have been proposed. Among them, the limitations of false positives and false negatives of the tests can be overcome by employing two independent tests that detect different antibodies or use different antigens or protocols, as discussed in the previous topics [173,185,186]. For example, even if high amounts of IgM are observed, indicating recent virus exposure, a standard of care molecular test should still be conducted to examine for a viral RNA presence. However, the application of the two tests of different categories could increase the costs associated with population testing, which is not interesting in economic terms, especially for developing countries. In these cases, it is important the existence of strategic measures for population testing, using tests that can corroborate to effective health policies and that can, to some extent, reduce costs associated to maintaining the public health system, since they can act in reducing hospitalization admission rates.

Currently, there is a diversity of quality and price for RDTs available on the market. This is mainly because, initially, the regulatory agencies, such as the FDA, did not require either formal approval or an emergency use authorization of a particular assay prior to it being used in a clinical setting [142]. Due to this, to minimize false negative results, it is recommended that researchers choose tests with high specificity (99.5% or higher) [209]. This measure is important because deregulation or misuse of these rapid tests can create an extra panic in society [33].

Thus, the perspectives associated with serological tests are associated with the improvement of the PoC tests already available, using technologies that allow for improving the analytical performance of the tests.

## 4. Nucleic Acid-Based Tests and Serological Tests: Practical Recommendations

**Figure 4:**
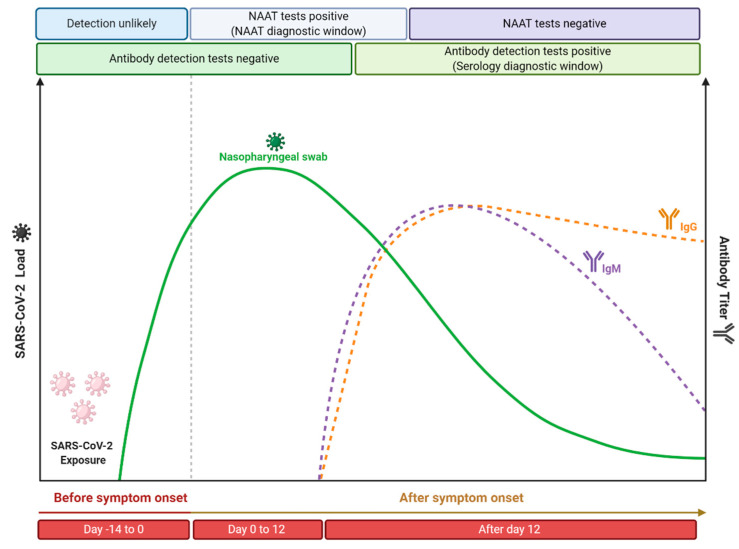
Time course of COVID-19 illness, considering the positivity variation over time in serological and nucleic acid-based tests for detection of SARS-CoV-2 infection. This figure was elaborated from the data contained in the work of Sethuraman et al. [213]. Created with BioRender.com.

As previously described, molecular and serological tests present different kinetics, since it depends on the capacity of viral replication (viral load) or on physiology antibody production, respectively (Figure 4). Currently, it is believed that the incubation period, which is the time of exposure and the onset of symptoms, is between four or five days [210]. In this period, the viral load gradually increases, while shortly after the onset of symptoms, it decreases over time [211]. Therefore, it is expected that molecular tests performed soon after the onset of symptoms will be positive, while as time progresses and, consequently, the viral load and infectiousness decrease, these tests will become negative. However, Sethuraman et al. [212] observed that the RT-qPCR test was able to produce positive results in the first three weeks after the onset of symptoms. This may be related to the test’s ability to check only for the presence of viral RNA and not the amount of viable viruses [213]. It is important to note that PCR positivity does not always correlate with the clinical severity of the disease, since cases have already been reported in which, even with a positive molecular test, the disease symptoms have been completely resolved [214]. The viral load of SARS-CoV-2 is normally defined through the cycle threshold (Ct) and, in a practical application, it is considered that a Ct value less than 40 is clinically reported as PCR positive [212]. Ct value is defined as the number of replication cycles in which the fluorescence of the sample exceeds a chosen threshold above the calculated background fluorescence [215]. In other words, the lower the Ct value obtained, the more the gene exists in the sample, which can represent a higher viral load [216]. Despite the recommendation to perform the RT-qPCR test on samples collected from the appearance of symptoms, since the viral load is higher [217], some studies indicate that the Ct value between symptomatic and asymptomatic patients is similar [218,219,220]. This indicates that even if the appearance of symptoms is important for the performance of molecular tests, since it is related to the increase in viral load, these tests can also be used in samples from asymptomatic patients, once the found Ct value can be similar. Another important point is that the timeline of PCR positivity is different depending on the type of specimen used. It has been shown that PCR positivity decreases more slowly in sputum and can still be positive even after nasopharyngeal swabs are negative [221]. Therefore, it is recommended that the molecular testes (especially the RT-qPCR technique) should be performed soon after the onset of symptoms from respiratory tract specimens, especially those obtained from the lower respiratory tract, to minimize the chances of false negative results.

In contrast, the application of serological tests is recommended from the second week since the onset of symptoms. Antibodies are more likely to be detected in serology assays after 12 or 14 days after the onset of the first symptoms [222], with IgG levels generally higher than IgM levels from about four weeks after the onset of symptoms [223]. More precisely, different studies suggest that 14 days after the onset of symptoms is a time period at which serological tests have high sensitivity that is able to replace nucleic acid amplification tests for the effective diagnosis of COVID-19 [224,225,226]. In addition, it is important to highlight that it has been demonstrated that more severe cases of COVID-19 have a late humoral response compared to mild/moderate infections [227], as well as high antibody titers have been shown to be associated with the severity of the disease [228]. Similar behavior was also found when neutralizing antibody titers were analyzed, where it was reported that asymptomatic patients do not produce a prolonged response [229], while patients with moderate to moderate symptoms produced robust responses against the SARS-CoV-2 spike protein [230]. Li et al. [228] observed the production of antibodies in adults reached their peak at 17 days after the onset of symptoms and then remained at a relatively high level for up to 50 days. Similar behavior was also reported by Roarty et al. [231], who demonstrated that antibody titers in children previously exposed to SARS-CoV-2 remained detectable up to 62 days after the onset of symptoms. Thus, it is generally expected that the performance of serological tests (such as the parameters of specificity, sensitivity, and accuracy) will improve according to the course of the illness.

## 5. Conclusions

The COVID-19 pandemic challenged the medical community across the globe in ways that could not have been predicted. In the absence of effective therapies, population testing is still the main tool to confront the COVID-19 pandemic. The search for an immediate response to the pandemic caused different regulatory agencies to release diagnostic kits to the consumer market at the beginning of the pandemic without a critical analytical validation. Due to this, this work showed that the studies available in the scientific literature showed that the main problem associated with molecular and serological tests for the detection of SARS-CoV-2 is the analytical performance, resulting in the release of false negative and false positive results.

Therefore, reducing the number of false negative test results is vital for determining quarantine measures and cohorts for hospitalized patients. To mitigate this problem, diagnosis that combines different techniques has been considered as an interesting alternative. However, this alternative is often not feasible in many countries and organizations due to the associated costs of using more than one technique for diagnosis. It is important to highlight that the use of any test methodology for population testing requires the investment of resources, logistical support, and the implementation of guidelines. In addition, planning on mass testing should be sustainable, as SARS-CoV-2 is expected to be in circulation for a long time. Even with resource restrictions, be they human or financial, it is essential that these costs be considered, since population-wide testing can reduce rates of abstention from work, hospitalization, mortality as well as other onerous measures. Different shortcomings have been exposed in this sector, particularly with regard to the complexity of the test execution steps, as well as the high cost of the associated equipment and materials, mainly related to molecular biology techniques. This has caused many countries to face problems related to the shortage of molecular diagnostic kits. Although no meta-analysis was performed in the evaluated studies, this systematic review would be helpful since the analyzed studies indicate that the big trade-off in diagnostics at present is between accuracy and throughput on the one hand and rapidity, simplicity, and availability on the other. Thus, the COVID-19 pandemic demonstrated the need for investment in this sector, which has become essential to combat outbreaks caused by emerging microorganisms.

## Figures and Tables

**Table 1:** World Health Organization list of testing protocols.

Country	Institute	Molecular Targets
China	China Center for Disease Control and Prevention (CDC)	Open Reading Frame (ORF) 1ab and N
Germany	Charité	RdRp, E, N
Hong Kong	Hong Kong University (HKU)	ORF1b-nsp14, N
Japan	National Institute of Infectious Diseases, Department of Virology III	Japan Pancorona and multiple targets, S
Thailand	National Institute of Health	N
United States	CDC	Two regions in N protein
France	Institut Pasteur	Two regions in RdRp

**Table 2:** Performance analyses of RT-qPCR kits and systems or platforms for the diagnosis of COVID-19.

Reference	Commercial Kit Name (Manufacturer)	System or Platform	Specimen Type	No. of Patients/Specimens	Gene(s) Target (Reference Method)	Main Findings and/or Conclusions
Visseaux et al. [83]	RealStare Severe Acute Respiratory Syndrome Coronavirus 2 (SARS-CoV-2) RT-PCR kit (Altona Diagnostic)	ABI 7500 real-time PCR system (Applied Biosystems)	Nasopharyngeal swabs	83	E and S	The RealStar^®®^ SARS-CoV-2 demonstrated a slightly higher sensitivity than the WHO recommended assay. Sensitivity: 97.8% Specificity: 97.3% No cross reaction with the other human coronaviruses
Yip et al. [84]	LightMix^®®^ Modular E-gene kit (Roche)	LightCycler 480 II Real-Time PCR System (Roche)	Nasopharyngeal aspirate	289	E	The LightMix^®®^ E-gene kit had similar sensitivity to the in-house assays. Sensitivity: 51.6% No cross reaction with the other human coronaviruses, metapneumovirus, rhinovirus, adenovirus, respiratory syncytial, influenza, and parainfluenza viruses
Nalla et al. [85]	BGI RT-PCR detection kit (BGI)	ABI 7500 real-time PCR system (Applied Biosystems)	Nasopharyngeal and oropharyngeal	375	ORF1ab	Specificity: 100% Sensitivity: variation according to the serial dilutions of RNA No cross-reactivity with other respiratory viruses
Szymczak et al. [86]	Xpert Xpress SARS-CoV-2 (Cepheid)	GeneXpert Infinity (Cepheid)	Stool	79	E and N2	Positive percent agreement between the Cepheid and Hologic assays was 93% (95% CI: 81.1–98.2%) and negative percent agreement was 96% (95% CI: 89–0.99%). No cross-reactivity with bacterial organisms was found in the gastrointestinal tract
	Hologic Panther Fusion (Hologic)	Panther Fusion System (Hologic)			ORF1a	
Pujadas et al. [87]	cobas SARS-CoV-2 RT-PCR test (Roche)	Cobas 6800 System (Roche)	Nasopharyngeal swabs	1006	ORF1/a and E	A Cohen’s kappa coefficient was calculated between the definitive results from the two platforms and was found to be 0.904 (95% CI, 0.875–0.933), suggesting almost perfect agreement between both platforms
	Laboratory-developed test (based on a modified CDC protocol)	LightCycler 480 II (Roche)			N1, N2, and N3	
Craney et al. [88]	cobas SARS-CoV-2 RT-PCR test (Roche)	Cobas 6800 System (Roche)	Nasopharyngeal swabs	389	ORF1/a and E	The overall percent agreement between the platforms was 96.4% (375/389). Cohen’s kappa analysis rated the strength of agreement between the two platforms as “almost perfect” (κ = 0.922; standard error, 0.051).
	Panther Fusion SARS-CoV-2 RT-PCR test (Hologic)	Panther Fusion System (Hologic)			ORF1ab	
	Xpert Xpress SARS-CoV-2 RT-PCR test (Cepheid)	-			E and N2	
Perng et al. [89]	BD MAX (BD)	BD Max Open System (BD)	Throat swab and sputum	400	E and RdRp	Concordant results were obtained for both assays in SARS-CoV-2 detection, showing 100% agreement
	Laboratory-developed test (based on Charité protocol [48])	Rotor-Gene Q (Qiagen)			ORF1ab and E	
van Kasteren et al. [90]	RealStar SARS-CoV-2 RT-PCR Kit 1.0 (Altona Diagnostics)	LightCycler 480 II (Roche)	Naso-and/or oropharyngeal swabs	22	E and S	PCR efficiency was ≥96% for all assays and the estimated LOD95 varied within a six-fold range None of the assays showed cross-reactivity with other respiratory (corona)viruses, except as expected for the SARS-CoV-1 E-gene
	Real-Time Fluorescent RT-PCR Kit for Detecting SARS-CoV-2 (BGI)				ORF1ab	
	VIASURE SARS-CoV-2 Real Time PCR Detection Kit (CerTest Biotec)				ORF1ab and N	
	RADI COVID-19 Detection kit (KH Medical)				RdRp and S	
	Coronavirus (COVID-19) (PrimerDesign)				RdRp	
	RIDA GENE SARS-CoV-2 RUO (R-Biopharm AG)				E	
	Allplex™ 2019-nCoV Assay (Seegene)				RdRp, N and E	
Iglói et al. [91]	RealStar SARS-CoV2 1.0 (Altona Diagnostics)	LightCycler 480 (Roche) and Quantstudio5 (Thermo- fisher Scientific)	SARS-CoV-2 cell-cultured virus stock		E, S	All RT-PCR kits included exhibited PCR efficiencies >90%, except for the Sentinel Diagnostics B E-gene assay (80%) Analytical sensitivity varied between 3.3 RNA copies to 330 RNA copies Only one assay (Powercheck 2019-nCoV) cross reacted with another human coronavirus (MERS).
	LightMix Sarbeco-E/SARS-CoV-2 RdRp (Tibmolbiol)				E, RdRp	
	Taqman 2019-nCoV Assay kit v1 (ThermoFisher)				ORF1ab, S, N	
	Detection Kit for 2019-nCoV (DAAN Gene)				ORF1ab, N	
	Powercheck 2019-nCoV (Kogene Biotech)				ORF1ab, E	
	2019-nCoV realtime multiplex RT-PCR (Liferiver)				ORF1ab, N	
	SARS-CoV2 fluorescent PCR (Maccura Biotechnology)				ORF1ab, E, N	
	Ridagene SARS-CoV2 (R-Biopharm)				E	
	2019-nCoV nucleic acid diagnostic kit (Sansure Biotech)				ORF1ab, N	
	STAT-NAT COVID19 B (Sentinel Diagnostics B)				E, RdRp, N	
	STAT-NAT COVID19 HK (Sentinel Diagnostics HK)				ORF1ab, N	
	SARS-CoV-2 Realtime PCR assay kit (XABT)				ORF1ab, E, N	
	RT PCR kit for detection of 2019-nCoV (Hecin Scientifi)				RdRp, N	
Bordi et al. [92]	Simplexa™ COVID-19 Direct assay (DiaSorin Molecular)	LIAISON MDX (DiaSorin Molecular)	Nasal and nasopharyngeal swabs	278	ORF1ab and S	Cross-reactive analysis performed in 20 nasopharyngeal swabs confirmed 100% of the clinical specificity of the assay Clinical performances of the Simplexa COVID-19 Direct assay were was compared to the Charité protocol [48]. Concordance analysis showed an “almost perfect” agreement in SARS-CoV-2 RNA detection between the two assays, being κ = 0.938; SE = 0.021; 95% CI = 0.896–0.980

**Table 3:** Analyses of different ELISA commercial kits for diagnosis of COVID-19.

Reference	Commercial Kit Name (Manufacturer)	No. of Patients/Samples	Country of the Test Population	Antibodies Detected	Days from Disease Onset	Main Findings and/or Conclusions
Brochot et al. [158]	Euroimmun SARS-CoV-2 IgG (Euroimmun)	168	France	IgG (recombinant S protein)	≥ 9	Considering hospitalized patients, all these assays showed a sensitivity of 100% from day 9 after the symptoms onset. However, the sensitivity was much lower for patients who did not require hospitalization for COVID-19 confirmed by PCR with 69% for the Euroimmun assay and 91.6% for the Wantai assay
	Wantai SARS-CoV-2 Ab ELISA (Beijing Wantai)			Total antibodies IgM and IgG (recombinant spike protein receptor binding domain (RBD))		
Kohmer et al. [159]	Euroimmun SARS-CoV-2 IgG (Euroimmun)	33	Germany	IgG (recombinant S protein)	5 to 18	The sensitivity was 58.8% for the Euroimmun assay and 70.6% for the Vircell assay on days 5–9 after confirmation by RT-qPCR. On the other hand, 10–18 days after confirmation, the sensitivity was 93.8% and 100% for the Vircell assay and Euroimmun assay, respectively
	Vircell COVID-19 ELISA IgG (Vircell)			IgG (recombinant N protein)		
Tuaillon et al. [160]	ID Screen SARS-CoV-2-N IgG (ID Vet)	58	France	IgG (recombinant N protein)	1 to ≥15	The commercial ELISAs demonstrated similar sensitivity of 86.7% with 80–100% specificity, depending on the day the samples were collected. However, IgG and IgA assays by the Euroimmun company suffered from a specificity below 90%
	SARS-CoV-2 IgA and IgG (Euroimmun)			Either IgA or IgG (recombinant subunit protein 1 (S1))		
Lassaunière et al. [161]	Wantai SARS-CoV-2 Ab ELISA (Beijing Wantai)	111	Denmark	Total antibodies IgM and IgG (recombinant spike protein RBD)	7 to ≥21	The diagnostic performance of the commercial assays analyzed may vary The results showed 100% specificity for the Wantai assay, 96% for the Euroimmun IgG assay, and 93% for the Euroimmun IgA assay, with sensitivities of 90%, 65%, and 90%, respectively
	Anti-SARS-CoV-2 IgG and IgA (Euroimmun)			Either IgA or IgG (recombinant subunit protein 1 (S1))		
Geurtsvan-Kessel et al. [162]	Wantai SARS-CoV-2 total Ig and IgM ELISAs (Beijing Wantai)	147	Netherlands	Total antibodies IgM and IgG (recombinant spike protein RBD) and IgM and (recombinant spike protein RBD)	>14	The Wantai assay was able to detect the total immunoglobulins against the receptor binding domain of SARS CoV-2 in different stages and severities of COVID-19
	Anti-SARS-CoV-2 IgG and IgA (Euroimmun)			Either IgA or IgG (recombinant subunit protein 1 (S1))		
Jääskeläinen et al. [163]	Anti-SARS-CoV-2 IgG and IgA (Euroimmun)	37	Finland	Either IgA or IgG (recombinant subunit protein 1 (S1))		The results showed a higher specificity of SARS-CoV-2 IgG ELISA (91.9%) than SARS-CoV-2 IgA ELISA (73.0%); therefore, it is not suggested to use the IgA assay for initial screening

**Table 4:** Analyses of different rapid diagnostic tests (RDT) commercial kits for the diagnosis of COVID-19.

Reference	Commercial Kit Name (Manufacturer)	No. of Patients/Samples	Country of the Test Population	Days from Disease Onset	Antibodies Detected	Main Findings and/or Conclusions
Cassaniti et al. [188]	VivaDiag COVID-19 IgM/IgG Rapid Test (VivaChek)	50	Italy	7 days	IgM and IgG	The majority of patients that diagnosed as positive for COVID-19 by RT-qPCR would have been identified as negative using only the rapid serological assay, leading to a misdiagnosis of COVID-19 disease
Pan et al. [189]	Diagnostic Kit for IgM/IgG Antibody to Coronavirus (SARS-CoV-2) (Lateral Flow) (Zhuhai Livzon Diagnositic)	105	China	1 to >15	IgM and IgG	The positive rates of IgG and IgM in the early stages are relatively low and gradually increase during disease progression, where the IgM-positive rate increased from 11.1% to 74.2% according to the progression of the disease, as well as the IgG-positive rate, which initially was 3.6% and increased to 96.8%
Shen et al. [190]	Test IgM/IgG SARS-CO-2 (Shanghai Outdo Biotech)	97	China	1 to >15	IgM and IgG	The kit assay for SARS-Cov-2 specific IgM/IgG antibody demonstrated 71.1% and 96.2% for the sensitivity and specificity, respectively, in this survey population, showing the potential for a useful rapid diagnosis test for COVID-19
Pérez-García et al. [191]	AllTest COV-19 IgG/IgM kit	163	Spain	1 to 17	IgM and IgG	The specificity found was 100% and the sensitivity of the test was 73.9% after 14 days from the onset of symptoms
Spicuzza et al. [192]	2019-nCoV IgG/IgM An- tibody Rapid Test Kit (Beijing Diagreat Biotechnologies)	37	Italy		IgM and IgG	The results reported suggest that the rapid IgG/IgM test was reliable in evidencing seroconversion as long as the testing was not performed <6 days before the symptom onset
Virgilio-Paradiso et al. [193]	SARS-CoV-2 rapid IgG-IgM VivaDiag Test (VivaChek)	191	Italy	1 to >15	IgM and IgG	In general, the performance of the test at the onset of symptoms was low: sensitivity of 30% and a specificity of 89% with respect to the standard assay but these performances improved after 8 days of symptom appearance. After 10 days of symptoms, the predictive value of the rapid serological test was higher than that of the standard assay
Demey et al. [194]	Commercial Name Not Informed (Biotime Biotechnology)	22	France	1 to 24	IgM and IgG	The median antibody detection time was between 8 and 10 days according to the evaluated kit. In general, all the tests showed a sensitivity of 60% to 80% on day 10, with the increase to 100% on day 15. A single cross-reaction was observed with other human coronavirus infections (HCoV-229E)
	Commercial Name Not Informed (Autobio Diagnostics)				IgM and IgG	
	Commercial Name Not Informed (ISIA BIO-Technology)				IgM and IgG	
	Commercial Name Not Informed (Biolidics)				IgM and IgG	
Serrano et al. [195]	Commercial Name Not Informed (Hangzhou Alltest Biotech)	152	Spain	1 to 28	IgM, IgG and IgG/IgM	In general, the test kits showed variable performances, with the specificity ranging from 88.3% to 100%. The overall results were better for the Guangzhou Wondfo Biotech manufacturer. An ELISA assay was also performed, and the values related to its performance included sensitivities for IgG and IgA of 81.5% and 93.1% and specificities of 100% and 80.6%, respectively. The authors suggested that commercial ELISA assays and LFI tests can be used as complementary tools in COVID-19 diagnosis
	Commercial Name Not Informed (Wuhan UNscience Biotechnology)				IgM, IgG and IgG/IgM	
	Commercial Name Not Informed (Guangzhou Wondfo Biotech)				IgM/IgG	
Mlcochova et al. [196]	COVIDIX 2019 SARS-CoV-2 IgG/IgM Test (COVIDIX Healthcare)	128	United Kingdom	1 to 28	IgG and IgM	Antibody detection by LFIA increased according to the progression of the disease, with 100% efficacy beyond the 9th day post-symptoms
	SureScreen SARS-CoV-2 IgG/IgM Test (SureScreen Diagnostics)				IgG and IgM	

## Data Availability

Data is contained within the article.
